# Nutrient deprivation and hypoxia alter T cell immune checkpoint expression: potential impact for immunotherapy

**DOI:** 10.1007/s00432-022-04440-0

**Published:** 2022-11-29

**Authors:** Maria Davern, Noel E. Donlon, Fiona O’Connell, Caoimhe Gaughan, Cillian O’Donovan, Jason McGrath, Andrew D. Sheppard, Conall Hayes, Ross King, Hugo Temperley, Michael MacLean, Christine Bulter, Anshul Bhardwaj, Jenny Moore, Claire Donohoe, Narayanasamy Ravi, Melissa J. Conroy, John V. Reynolds, Joanne Lysaght

**Affiliations:** 1 Cancer Immunology and Immunotherapy Group, Department of Surgery, Trinity St. James’s Cancer Institute, Trinity Translational Medicine Institute, Trinity College, St. James’s Hospital Campus, Dublin 8, Ireland; 2 Department of Surgery, Trinity St. James’s Cancer Institute, Trinity Translational Medicine Institute, Trinity College Dublin, St. James’s Hospital, Dublin, Ireland

**Keywords:** Glucose deprivation, Hypoxia, Oesophageal adenocarcinoma, Immune checkpoints, Tumour microenvironment

## Abstract

**Aim:**

Use of immune checkpoint blockade to enhance T cell-mediated immunity within the hostile tumour microenvironment (TME) is an attractive approach in oesophageal adenocarcinoma (OAC). This study explored the effects of the hostile TME, including nutrient deprivation and hypoxia, on immune checkpoint (IC) expression and T cell phenotypes, and the potential use of nivolumab to enhance T cell function under such conditions.

**Methods and Results:**

ICs were upregulated on stromal immune cells within the tumour including PD-L2, CTLA-4 and TIGIT. OAC patient-derived PBMCs co-cultured with OE33 OAC cells upregulated LAG-3 and downregulated the co-stimulatory marker CD27 on T cells, highlighting the direct immunosuppressive effects of tumour cells on T cells. Hypoxia and nutrient deprivation altered the secretome of OAC patient-derived PBMCs, which induced upregulation of PD-L1 and PD-L2 on OE33 OAC cells thus enhancing an immune-resistant phenotype. Importantly, culturing OAC patient-derived PBMCs under dual hypoxia and glucose deprivation, reflective of the conditions within the hostile TME, upregulated an array of ICs on the surface of T cells including PD-1, CTLA-4, A2aR, PD-L1 and PD-L2 and decreased expression of IFN-γ by T cells. Addition of nivolumab under these hostile conditions decreased the production of pro-tumorigenic cytokine IL-10.

**Conclusion:**

Collectively, these findings highlight the immunosuppressive crosstalk between tumour cells and T cells within the OAC TME. The ability of nivolumab to suppress pro-tumorigenic T cell phenotypes within the hostile TME supports a rationale for the use of immune checkpoint blockade to promote anti-tumour immunity in OAC.

**Graphical abstract:**

Study schematic: (A) IC expression profiles were assessed on CD45^+^ cells in peripheral whole blood and infiltrating tumour tissue from OAC patients in the treatment-naïve setting. (B) PBMCs were isolated from OAC patients and expanded ex vivo for 5 days using anti-CD3/28 + IL-2 T cell activation protocol and then co-cultured for 48 h with OE33 cells. T cell phenotypes were then assessed by flow cytometry. (C) PBMCs were isolated from OAC patients and expanded ex vivo for 5 days using anti-CD3/28 + IL-2 T cell activation protocol and then further cultured under conditions of nutrient deprivation or hypoxia for 48 h and T cell phenotypes were then assessed by flow cytometry. Key findings: (A) TIGIT, CTLA-4 and PD-L2 were upregulated on CD45^+^ immune cells and CTLA-4 expression on CD45^+^ cells correlated with a subsequent decreased response to neoadjuvant regimen. (B) Following a 48 h co-culture with OE33 cells, T cells upregulated LAG-3 and decreased CD27 co-stimulatory marker. (C) Nutrient deprivation and hypoxia upregulated a range of ICs on T cells and decreased IFN-γ production by T cells. Nivolumab decreased IL-10 production by T cells under nutrient deprivation-hypoxic conditions.

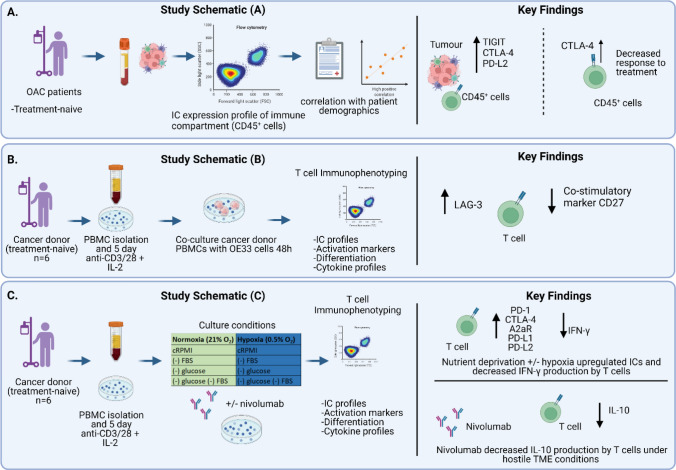

**Supplementary Information:**

The online version contains supplementary material available at 10.1007/s00432-022-04440-0.

## Introduction

Oesophageal adenocarcinoma (OAC) is the predominant subtype of oesophageal cancer in the Western world and its incidence is continuing to increase rapidly (Pera et al. 2005). The cellular and physiological composition of the tumour microenvironment (TME) plays a pivotal role in the development and progression of OAC, including dictating the response to the current standards of care (Davern et al. 2021a). The TME imposes profound metabolic restrictions on anti-tumour T cells, and understanding these insights is important for informing immunotherapeutic anti-cancer strategies (DePeaux and Delgoffe 2021). Therapeutic approaches such as those targeting metabolic restrictions, including low glucose levels and hypoxia have shown promise as combination therapies for different types of cancer (Guo et al. 2019). Both nutrient deprivation and hypoxia have a profound impact on the cellular composition of the TME—subsequently promoting or hindering anti-tumour immune responses. Hypoxia profoundly alters immune cell phenotypes, in particular the myeloid compartment, comprising of macrophages and myeloid-derived suppressor cells, which cooperatively promote immune evasion, tumour cell survival and metastatic dissemination (King et al. 2021). Tumour hypoxia promotes the recruitment of regulatory T cells through induction of the chemokine CC-chemokine ligand 28 which, in turn, promotes tumour tolerance and angiogenesis (Facciabene et al. 2011). In addition, cancer cells grow rapidly, outcompeting anti-tumour immune cells for essential nutrients and producing metabolic by-products, such as lactate which are toxic to immune cells, resulting in a both a nutrient starved and acidic hostile environment for anti-tumour immune cells (Singer et al. 2018).

The TME is often described as a glucose deprived environment, attributed to a poorly vascularised TME and cancer cells sequestering glucose for glycolysis to facilitate tumour progression (Park et al. 2004). These distinct metabolic pathways in tumour cells cause functional impairment in immune cells and contribute to immune evasion (Park et al. 2004). Glucose is also an essential nutrient for the metabolic demands and function of anti-tumour immune cells, particularly effector T cells (Yin et al. 2019). Glucose is an essential nutrient which plays an important role during the early stages of T cell activation in regulating T cell differentiation and maintaining activation states (Yin et al. 2019). T cell activation involves a dramatic increase in nutrient uptake and depletion of glucose or glutamine in cell culture media during the early stages of T cell activation inhibits T cell expansion, cytokine production and suppresses pro-inflammatory T cell differentiation (MacPherson et al. 2018). Conversely, regulatory T cells thrive in a glucose-depleted environment, as these cell types predominantly rely on fatty acid oxidation to fulfil their energy demands (Villa et al. 2021).

Metabolic reprogramming in T cells during their activation and differentiation have led to an emerging concept of “immunometabolism” (Giannone 2020). Considering the recent success of cancer immunotherapy in the treatment of several cancer types, increasing research efforts are elucidating alterations in metabolic profiles of cancer and immune cells in the setting of cancer progression and immunotherapy (Mockler et al. 2014). Therefore, immunometabolism is a key factor in regulating immune responses within the tumour. Immune checkpoint blockade (ICB), in particular programmed death-1 (PD-1) blockade, promotes the metabolic fitness of exhausted immune cells, reinvigorating an exhausted phenotype and enhancing anti-tumour effector T cell responses (Kazemi et al. 2021). However, a substantial number of OAC patients possess tumours refractory to ICB (Power et al. 2020). It is thought that concurrent targeting of immune checkpoints (ICs) and targeting immunometabolism pathways may have a greater effect in restoring effector functions to exhausted T cells (Kazemi et al. 2021). However, more in-depth research is required to study how the nutrient depleted and hypoxic TME might shape immune T cell function and alter responses to ICB in the context of OAC.

Therefore, this study investigates the direct effect of nutrient deprivation (serum deprivation and glucose deprivation) and hypoxia on the function of OAC patient-derived T cells, in particular the expression profile of ICs. Upregulation of inhibitory IC ligands on tumour cells and stromal cells that bind to inhibitory receptors on immune cells is key mechanism of immune suppression and represents a significant barrier for induction of effective anti-tumour immune responses (Toor et al. 2020). The effect of secreted mediators from OAC patient-derived PBMCs cultured under nutrient deprivation and hypoxia on IC expression profiles of OAC tumour cells is also assessed. This will provide a greater insight into how the crosstalk between immune cells and tumour cells under the physiological conditions of the TME alters IC expression profiles of OAC cells. Importantly, the ability of nivolumab to promote anti-tumour T cell-mediated immunity under conditions reflective of the hostile TME is also explored. Collectively, these findings will help guide the clinical development of rational immunotherapeutic strategies to improve immune responses within the inhospitable TME for treating OAC patients.

## Methods

### Ethical approval

Ethical approval was granted from the St. James’s Hospital/AMNCH Ethical Review Board. All samples were collected with prior informed written consent for sample and data acquisition from patients attending St. James’s Hospital or from healthy donors. This study was carried out in accordance with the World Medical Association’s Declaration of Helsinki guidelines on medical research involving human subjects. Patient samples were pseudonymised in line with GDPR and data protection policies to protect the privacy and rights of the patients.

### Specimen collection

All patients involved in this study were enrolled from 2018 to 2020. Treatment-naïve whole blood and tumour tissue biopsies were obtained from OAC patients undergoing endoscopy at St. James’s Hospital at time of diagnosis prior to initiation of chemotherapy or radiotherapy. The group consisted of 16 males and 6 females, with an average age of 66.4 years. The patient demographics are detailed in Table 1.

**Table 1:** Patient demographic

Age (years)	66.4
Sex ratio (M:F)	16:6
Diagnosis (no. patients)	
OGJ	11
OAC	11
Clinical tumour stage (no. patients)	
T0	0
T1	2
T2	6
T3	14
T4	0
Clinical nodal status (no. patients)	
Positive	11
Negative	11

### OAC tumour tissue digestion

Biopsies were enzymatically digested to perform OAC cell phenotyping as previously described in Davern et al. (2021b). Briefly, tissue was minced using a scalpel and digested in collagenase solution (2 mg/ml of collagenase type IV (Sigma) in Hanks Balanced Salt Solution (GE healthcare) supplemented with 4% (v/v) foetal bovine serum) at 37 °C and 1,500 rpm on an orbital shaker. Tissue was filtered and washed with FACs buffer (PBS containing 1% foetal bovine serum and 0.01% sodium azide). Cells were then stained for flow cytometry.

### Cell culture

Treatment-naïve OAC donor PBMCs (*n* = 8) were isolated from whole blood using Ficoll-Paque (GE healthcare) density gradient centrifugation and expanded with plate bound anti-CD3 (10 μg/ml, Biolegend, USA), anti-CD28 (10 μg/ml, Ancell, USA) and recombinant human IL-2 (Immunotools, Germany) for 5 days, followed by 24 h culture of PBMCs in cRPMI, serum-free RPMI, glucose-free RPMI or dual glucose-free and serum-free RPMI under normoxic or hypoxic conditions (0.5% O_2_) in the absence or presence of nivolumab (10 μg/ml) at 37 °C 5% CO_2_. Following this 6-day activation up to 85–90% of the lymphocyte population comprise of CD3^+^ T cells.

The OE33 cell line was established from a poorly differentiated stage IIA adenocarcinoma of the lower oesophagus (Barrett’s metaplasia) of a 73-year-old female patient and was purchased from the European Collection of Cell Cultures and grown in RPMI 1640 medium with 2 mM L-glutamine (Gibco) and supplemented with 1% (v/v) penicillin–streptomycin ((P/S) 50 U/ml penicillin 100 μg/ml streptomycin) and 10% (v/v) foetal bovine serum (Gibco) and maintained in a humidified chamber at 37 °C 5% CO_2_. Cell lines were tested regularly to ensure mycoplasma negativity.

### Nutrient deprivation and hypoxia treatment and co-culture of OAC donor PBMCs with OE33 cells

5-day expanded PBMCs were cultured for an additional 24 h in complete RPMI (cRPMI, 10% FBS, 1% P/S), serum-free RPMI (0% FBS, 1% P/S), glucose-free RPMI (Gibco, 10% FBS, 1% P/S), dual glucose-free and serum deprived RPMI (Gibco, 0% FBS, 1% P/S) under normoxic conditions (37 °C, 5% CO_2,_ 21% atmospheric O_2_) or hypoxic conditions (37 °C, 5% CO_2,_ 0.5% O_2_) using the H35 Don Whitley hypoxia station. PBMCs were then harvested for flow cytometry staining.

OE33 cells were seeded at a density of 1 × 10^4^ cells/100 μl in cRPMI in a flat-bottomed 96-well plate and left to adhere overnight. Following 24 h, 5-day expanded OAC patient-derived PBMCs were cultured alone or co-cultured with OE33 cells at a ratio of 5:1 (PBMCs:OE33 cells) for 48 h at 37 °C 5% CO_2_. PBMCs were then harvested for flow cytometry staining.

### Generation of OAC donor lymphocyte supernatant and co-culture with OE33 cells

Treatment-naïve OAC donor PBMCs were isolated from whole blood using Ficoll-Pacque (GE healthcare) and density gradient centrifugation, expanded for 5 days (using above anti-CD3/28 and IL-2 expansion protocol) and cultured for an additional 24 h under nutrient deprivation ± normoxic/hypoxic conditions (as described above). The supernatant was harvested and stored at − 80 °C for later use.

OE33 cells were seeded at a density of 1 × 10^4^ cells/100 μl in cRPMI in a flat-bottomed 96-well plate and left to adhere overnight. The media was replaced with 100 μl of cRPMI or 100 μl of 1 in 2 diluted supernatant that was collected from OAC donor PBMCs that had been cultured under hypoxia ± nutrient deprivation and cultured for 24 h at 37 °C 5% CO_2_.

### Flow cytometry staining

Fluorochrome-conjugated antibodies were added to 100 µl blood at pre-optimized concentrations and incubated for 15 min at room temperature in the dark. Red cells were lysed using red blood cell lysing solution (Biolegend, USA), according to manufacturer’s recommendations and cells were washed twice with FACs buffer and stained with zombie aqua viability dye (Biolegend, USA). Cells were fixed for 15 min in 1% paraformaldehyde solution (Santa Cruz Biotechnology, USA) prior to flow cytometric analysis.

Tumour tissue biopsies, healthy donor PBMCs or OAC donor PBMCs were stained with zombie aqua viability (Biolegend, USA) dye. Antibodies used for staining included ICOS-PE-eFluor610, LAG-3-FITC, CD160-PerCPCy5.5, PE-1-PE/Cy7, TIGIT-PE/Cy7, CD45RA-PE/Cy7, CD45RO-BV510, CD3-APC, CD3-PerCP, CD4-BV510, CD4-APC (Biolegend, USA), CD69-PE, CD62L-FITC, CD8-BV421 (BD Biosciences, USA), CD27-APC-eFluor780 (eBioscience, USA), TIM-3-AF647, CTLA-4-PE/Cy5, KLRG-1-APC, PD-L1-FITC, PD-L2-PE (BD Bioscience, USA), A2aR-PE (Bio-techne, USA). PBMCs were resuspended in FACs buffer and acquired using BD FACs CANTO II (BD Biosciences) using Diva software and analysed using FlowJo v10 software (TreeStar Inc.). Gating strategy on the lymphocyte population to assess T cell expression profiles of ICs is shown in Figure S1. Gating strategy on the lymphocyte population to assess T cell activation marker expression and T cell differentiation status to differentiate between naïve, central memory, effector memory and terminally differentiated effector memory T cells is shown in Figure S2.,

For intracellular cytokine staining PBMCs were treated with PMA (10 ng/ml) and ionomycin (1 µg/ml) for the last 4 h of the incubation. For the last 3 h of the incubation PBMCs were treated with brefeldin A (10 µg/ml, eBiosciences). Cells were harvested, washed in FACs buffer and intracellular cytokines were assessed using a Fixation/Permeabilisation kit (BD Biosciences), as per manufacturer’s recommendations. Cells were stained with cell surface antibodies (CD8-BV421, CD3-APC or CD3-PerCP, CD4-PerCP, CD4-APC or CD4-BV510 (Biolegend, USA)) washed, permeabilised, and then stained for intracellular cytokines: IFN-γ-BV510, IL-17A-FITC, Granzyme B-PE/Cy7, Perforin-FITC-BV510 (Biolegend, USA) and TNF-α-APC (BD Biosciences, USA). Cells were resuspended in FACs buffer and acquired using BD FACs CANTO II (BD Biosciences). Gating strategy on the lymphocyte population to assess T cell cytokine production is shown in Figure. S3.

### Statistical analysis

Data were analysed using GraphPad Prism version 5 (GraphPad Prism, San Diego, CA, USA) software and was expressed as mean ± SEM. Statistical differences between treatments within cancer donors or within healthy donors were analysed using paired non-parametric t test and statistical differences between treatments between healthy donors and cancer donors were analysed using unpaired non-parametric t tests. Statistical significance was determined as p ≤ 0.05. Spearman correlations were performed to analyse correlation data between clinical characteristics and flow data and visualised using the R package ‘corrplot’.

## Results

### TIGIT, CTLA-4 and PD-L2 immune checkpoints were upregulated on CD45^+^ cells within the OAC TME

**Fig. 1:**
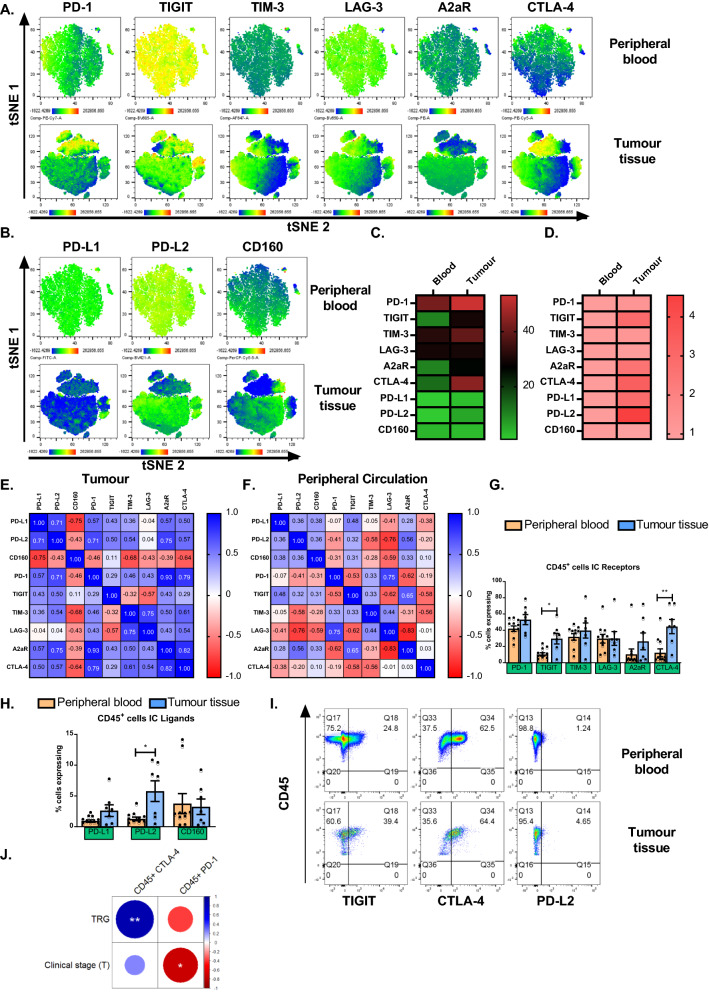
TIGIT, CTLA-4 and PD-L2 are expressed at significantly higher levels on the surface of CD45^+^ cells infiltrating tumour tissue compared with peripheral circulation and frequencies of CD45^+^CTLA-4^+^ circulating cells positively correlated with a subsequent poor pathological response to treatment. CD45^+^ cells were screened for the surface expression of IC receptors PD-1, TIGIT, TIM-3, LAG-3, A2aR and CTLA-4 and ligands PD-L1, PD-L2 and CD160 in peripheral whole blood (*n* = 10) and infiltrating OAC pre-treatment tumour biopsies (*n* = 7) by flow cytometry. tSNE plots are shown depicting the spatial distribution and expression of each IC receptor (**A**) and ligand (**B**) on CD45^+^ cells in peripheral circulation and infiltrating tumour tissue. Heat maps summarising the relative expression levels (**C**) and fold change (**D**) of IC proteins on CD45^+^ cells in peripheral blood versus tumour tissue. (**E**) and (**F**) show the correlation values between IC expression in peripheral blood and tumour tissue, respectively. Graphical depiction of IC receptor and ligand expression on CD45^+^ cells in peripheral blood and tumour tissue is shown in (**G**) and (**H**), respectively. (**I)** includes representative dot plots of the IC proteins TIGIT, CTLA-4 and PD-L2 which are significantly upregulated on tumour-infiltrating CD45^+^ cells compared with those in circulation. Mann Whitney test *p < 0.05. (**J)** Corrogram depicting the significant correlations between circulating CD45^+^ cells expressing ICs and clinical parameters in treatment-naïve OAC patients. Spearman correlation *p ≤ 0.05. Mandard tumour regression grade (*TRG*) clinical tumour (*T*) stage determined by PET/CT

The TME is a well-characterised inhospitable environment for immune cells, frequently nutrient deprived and hypoxic, which has a profound effect on the immune infiltrate typically promoting an immunosuppressive phenotype. To provide insight into the direct effects of the OAC TME on immune checkpoint (IC) expression, a range of IC proteins was assessed on the surface of CD45^+^ cells in the tumour tissue and in circulation (Fig. 1).

There was a significantly higher percentage of CD45^+^ cells within the tumour tissue expressing TIGIT compared with circulating CD45^+^ cells (Fig. 1A and 1G). Similarly, CTLA-4 was expressed on a significantly higher percentage of CD45^+^ cells in the tumour tissue compared with circulation (Fig. 1A and 1G). In addition, there was a significantly higher percentage of CD45^+^ cells within the tumour tissue expressing PD-L2 compared with circulating CD45^+^ cells (Fig. 1B and 1H).

IC expression on circulating and tumour-infiltrating immune cells has been shown to possess prognostic and predictive value of treatment response in several cancer types including OAC. Therefore, we investigated if the frequency of circulating or tumour-infiltrating CD45^+^ cells expressing ICs correlated with patient demographics or clinical features of the tumour, including treatment response, tumour stage and adverse features of the tumour, which have been shown to predict poor responses to current standards of care and survival in OAC patients in a study by Donlon et al. (2020), and include perineural invasion and lymphovascular invasion. The frequency of circulating CD45^+^CTLA-4^+^ cells positively correlated with a subsequent poor response to neoadjuvant treatment determined by the Mandard tumour regression grade (TRG) scoring system (Fig. 1J). In addition, the frequency of circulating CD45^+^PD-1^+^ cells negatively correlated with advanced tumour stage determined by PET/CT (Fig. 1J).

### OAC patient lymphocytes co-cultured with OE33 cells significantly upregulated LAG-3, altered T cell activation and cytokine profiles

Cancer cells grow rapidly within the TME producing waste by-products such as lactate, which adversely affects anti-tumour T cell function and can skew T cell phenotypes to a pro-tumour regulatory T cell. In addition, rapidly dividing cancer cells outcompete anti-tumour immune cells within the TME for essential nutrients such as glucose, amino acids and glutamine, which also significantly hinders the function of anti-tumour immune populations. Therefore, to ascertain the direct effect of cancer cell competition on T cell function, OE33 OAC cells were co-cultured with OAC donor PBMCs, and T cell phenotypes were subsequently assessed.

**Fig. 2:**
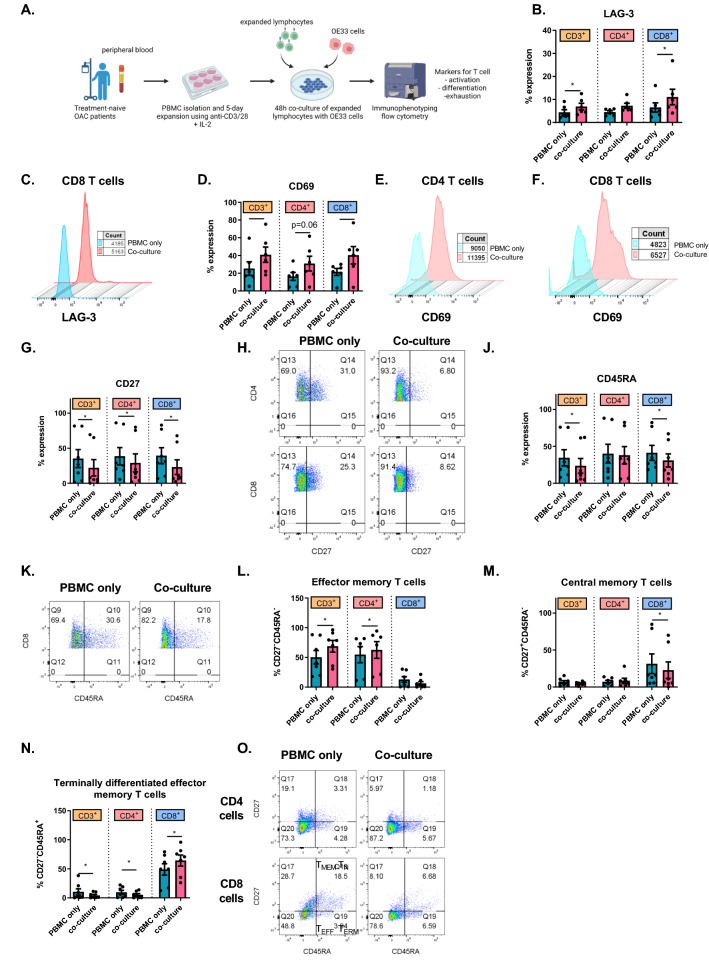
Co-culturing OE33 OAC cells with OAC donor PBMCs upregulates LAG-3 and alters the activation status of T cells. (**A**) Schematic of experimental setup. OE33 cells were cultured for 48h in the absence or presence of PBMCs (OE33: PBMCs, 1:2) isolated from OAC patients (*n* = 7) that were pre-activated for 5 days with plate bound anti-CD3/28 and IL-2 prior to culture. CD3^+^, CD3^+^CD4^+^ and CD3^+^CD8^+^ cells were then stained with a zombie viability dye and the expression of inhibitory immune checkpoint receptor LAG-3 (**B**), T cell activation markers CD69 (**D**), CD27 (**G**) and CD45RA (**J**), frequencies of effector memory (CD45RA^-^CD27^−^) (**L**), (central memory (CD45RA^-^CD27^+^) (**M**) and terminally differentiated effector memory (CD45RA^+^CD27^−^) (**N**) T cells was assessed via flow cytometry. Paired, non-parametric t test. Expression presented as percentage ± SEM on live cells. Only data with significant changes are shown and non-significant data is shown in Figure S5. Representative histograms and dot plots are shown for LAG-3 (**C**), CD69 (**E**–**F**), CD27 (**H**), CD45RA (**K**) and T cell differentiation status (**O**)

LAG-3 was significantly upregulated on CD8^+^ T cells co-cultured with OE33 cells compared with PBMCs cultured alone for 48 h (Fig. 2B and C). There was no significant alteration to the expression profile of PD-1, TIGIT, TIM-3, A2aR, CTLA-4, PD-L1, PD-L2 or CD160 on the surface of T cells following a 48 h co-culture with OE33 cells compared with PBMCs cultured alone (Figure S4).

Furthermore, CD69 was significantly upregulated on CD8^+^ T cells co-cultured with OE33 cells compared with PBMCs cultured alone for 48 h (Fig. 2D–F). However, CD27 was significantly decreased on CD4^+^ T cells and CD8^+^ T cells co-cultured with OE33 cells compared with PBMCs cultured alone for 48 h (Fig. 2G and H). Similarly, CD45RA was significantly decreased on the surface of CD8^+^ T cells following co-culture with OE33 cells compared with PBMCs cultured alone (Fig. 2J and K). There was no significant change in the expression of CD62L or CD45RO (Figure S5).

Interestingly, changes in T cell differentiation state were also observed following co-culture with OE33 cells. There was a significant increase in the percentage of effector memory CD4^+^ T cells compared with PBMCs cultured alone for 48 h (Fig. 2L and O). Furthermore, there was a significant decrease in the percentage of central memory CD8^+^ T cells (Fig. 2M and O) and a significant increase in the frequency of terminally differentiated effector memory CD8^+^ T cells following co-culture with OE33 cells compared with PBMCs that were cultured alone for 48 h (Fig. 2N and O).

There was no significant difference in the frequency of T cells producing of IL-17A/F, IFN-γ, TNF-α, IL-4 and IL-10 cytokines following a 48 h co-culture with OE33 cells compared with PBMCs cultured alone (Figure S6.).

### The secretome from OAC PBMCs upregulates PD-L1 and PD-L2 on the surface of OE33 cells

**Fig. 3:**
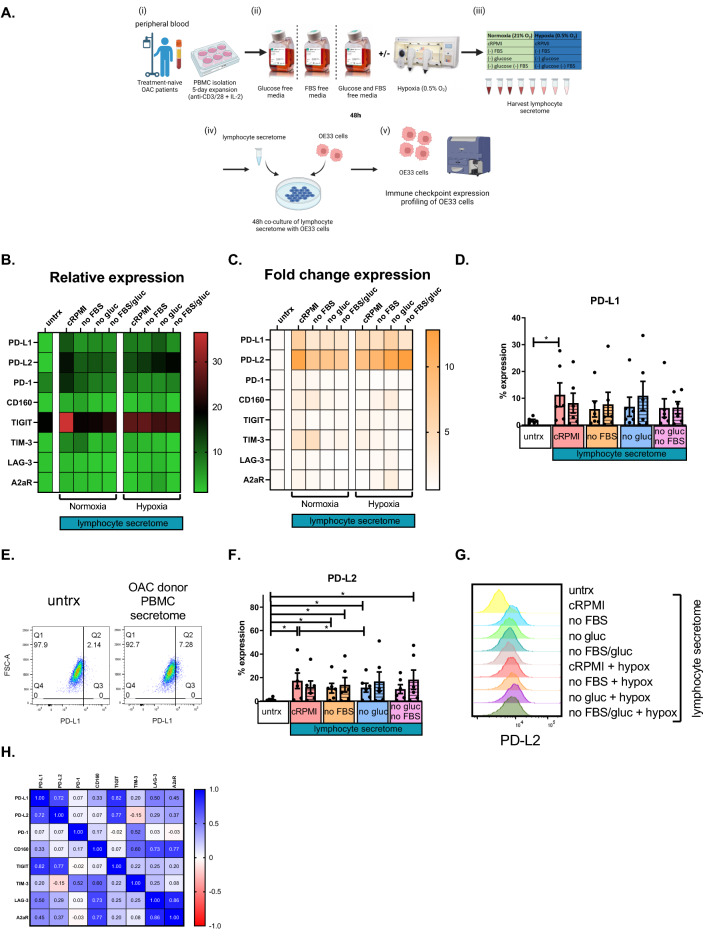
OAC patient-derived PBMC secretome significantly increased the expression of PD-L1 and PD-L2 on OE33 OAC cells under full nutrient and normoxic conditions. (**A**) Schematic of experimental setup. PBMCs were isolated from peripheral blood of treatment-naïve OAC patients (*n* = 6) and expanded for 5 days in the presence of plate bound anti-CD3/anti-CD28 and recombinant human IL-2. Following a 5-day expansion, PBMCs were cultured for an additional 48h under nutrient deprivation (FBS deprived or glucose deprived), hypoxia (0.5% O_2_) and combined nutrient deprivation hypoxic conditions, and the soluble secretome was harvested and cultured with OE33 cells using a 1 in 2 dilution for 24 h. OE33 cells were then stained with a zombie viability dye and antibodies specific for a range of ligands (PD-L1, PD-L2 and CD160) and IC receptors (PD-1, TIGIT, TIM-3, LAG-3 and A2aR) and expression was assessed by flow cytometry. Only significant data shown in graphs. Heat maps that summarise the effects of the lymphocyte secretome on the relative expression (**B**) and fold change (**C**) of ICs on the surface OE33 cells are also shown. PD-L1 (D-E) and PD-L2 (F-G) expression by OE33 cells following culture with OAC patient-derived PBMC secretome under a range of TME conditions. Undashed bars (left) represent normoxic conditions and dashed bars (right) represent hypoxic conditions. (**H**) Correlation matrix displaying the correlation values for IC expression on OE33 cells. Paired, non-parametric *t* test. Expression presented as percentage ± SEM on live cells

The nutrient deprived and hypoxic TME has a substantial effect not only on the phenotype of T cells but also on the T cell secretome, which could in turn alter IC expression by cancer cells and facilitate IC-mediated immune escape. Therefore, PBMCs from OAC patients were expanded and cultured under nutrient deprivation, hypoxia or both for 48 h after which the lymphocyte secretome was harvested and used to treat OE33 cells for 48 h. This experimental set up set out to determine if lymphocytes cultured under conditions reflective of the TME could upregulate ICs on the surface of OE33 cells, which could help propagate the TME-induced immune suppression (Fig. 3A). Figure 3B and C depicts heat maps which summarise the effects of the lymphocyte secretome on the relative expression and fold change of ICs on the surface OE33 cells. The secretome from OAC patient-derived T cells cultured in cRPMI significantly upregulated PD-L1 and PD-L2 on the surface of OE33 cells in vitro compared with untreated OE33 cells (Fig. 3D and E and F and G). Interestingly, the altered secretome of lymphocytes that had been cultured under serum deprivation, dual serum deprivation and hypoxia, glucose deprivation, dual glucose deprivation and hypoxia and dual glucose and serum deprivation in combination with hypoxia significantly upregulated PD-L2 on the surface of OE33 cells in vitro compared with untreated OE33 cells (Fig. 3F and G). There was no significant change in the expression of other ICs on the surface of OE33 cells following treatment with the lymphocyte secretome (CD160, PD-1, TIGIT, TIM-3, LAG-3, A2aR)(data not shown). In addition, the expression of ICs on the surface of OE33 cells positively correlated with each other (Fig. 3H). Overall, the secretome from OAC patient-derived PBMCs cultured in cRPMI had the greatest effect in upregulating PD-L1, while a combination of TME conditions upregulated PD-L2 on the surface of OE33 cells in vitro.

### Combination hypoxia and glucose deprivation upregulates PD-1, CTLA-4, PD-L1 and PD-L2 on the surface of OAC patient-derived T cells

Given the findings demonstrating that OAC donor lymphocytes co-cultured with OE33 cells significantly upregulated LAG-3 on the surface of T cells, we sought to investigate the direct effect of nutrient deprivation, including glucose deprivation and serum deprivation, and hypoxia on the IC expression profile of OAC donor lymphocytes. Cancer cells outcompete T cells for nutrients and oxygen within the TME resulting in a nutrient-deprived, hypoxic inhospitable microenvironment for T cells. Studies have demonstrated that hypoxia upregulates PD-L1 on the surface of tumour cells. As there is a wide range of immune checkpoint inhibitors (ICIs) currently under clinical development that target a wide spectrum of ICs, it is therefore important to elucidate the effects of the OAC TME on T cell IC expression. However, little is known about the effect of nutrient deprivation or hypoxia on the IC expression profile of OAC derived T cells. These findings will help guide the rationale selection of ICIs to reinvigorate T cells within this inhospitable environment.

**Fig. 4:**
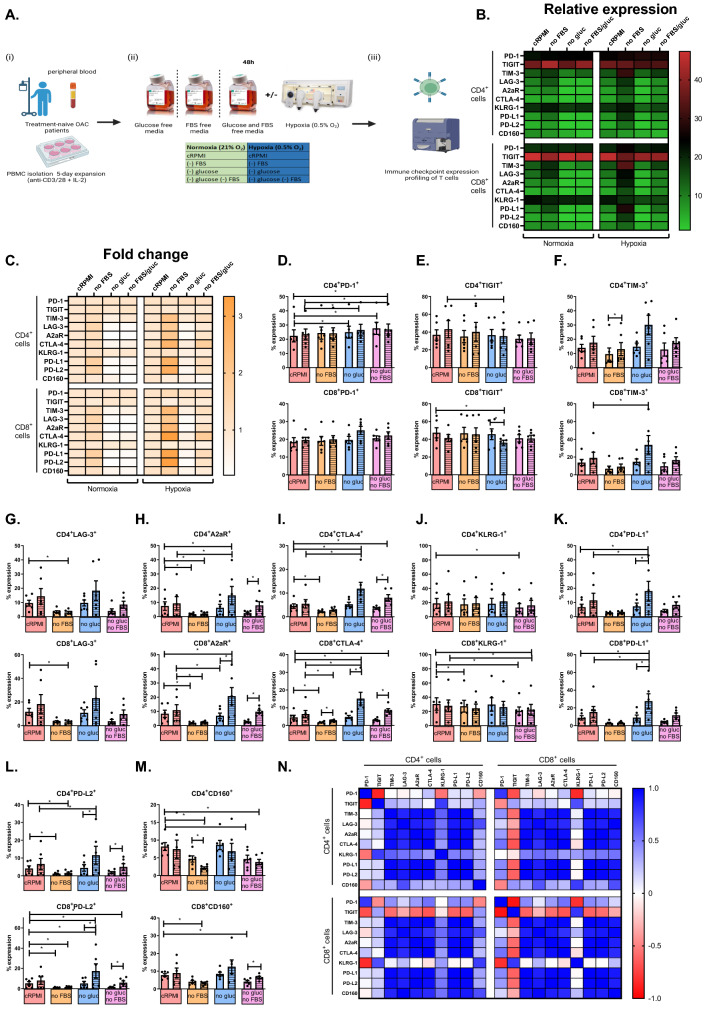
Combination hypoxia and glucose deprivation upregulated PD-1, CTLA-4, A2aR, PD-L1 and PD-L2 on the surface of OAC patient-derived T cells. **A** Schematic of experimental setup. PBMCs were isolated from peripheral blood of treatment-naïve OAC patients (*n* = 6) and expanded for 5 days in the presence of plate bound anti-CD3/anti-CD28 and recombinant human IL-2. Following a 5-day expansion, PBMCs were cultured for 48h under nutrient deprivation (FBS deprived or glucose deprived), hypoxia (0.5% O_2_) and combined nutrient deprivation and hypoxic conditions. CD3^+^CD4^+^ and CD3^+^CD8^+^ cells were stained with a zombie viability dye and antibodies specific for a range of inhibitory immune checkpoint receptors (PD-1, TIGIT, TIM-3, LAG-3, A2aR, CTLA-4 and KLRG-1 (**D**–**J**)) and inhibitory immune checkpoint ligands (PD-L1, PD-L2 and CD160 (**K**–**M**)) and expression was assessed by flow cytometry. Undashed bars (left) represent normoxic conditions and dashed bars (right) represent hypoxic conditions. Heat maps that summarise the effects hypoxia, nutrient deprivation and a combination of both on the relative expression (**B**) and fold change (**C**) IC expression profile of CD4^+^ and CD8^+^ cells are also shown. **N** Correlation matrix displaying the correlation values for IC expression on CD4^+^ and CD8^+^ T cells. Paired, non-parametric *t* test. Expression presented as percentage + SEM on live cells

Hypoxia treatment alone did not significantly affect the expression of ICs on the surface of OAC patient-derived T cells (Fig. 4). However, glucose deprivation significantly increased the expression of PD-1 on the surface of CD4^+^ T cells compared with cells cultured in cRPMI (Fig. 4). In contrast, serum deprivation significantly decreased A2aR, CTLA-4 and PD-L2 expression on the surface of CD4^+^ T cells compared with cells cultured in cRPMI (Fig. 4H, I and L). Similarly, serum deprivation significantly decreased A2aR, CTLA-4, KLRG-1 and PD-L2 expression on the surface of CD8^+^ T cells compared with cells cultured in cRPMI (Fig. 4H, I and J).

Dual hypoxia and serum deprivation significantly decreased LAG-3, A2aR and CD160 on the surface of CD4^+^ T cells compared with cells cultured in cRPMI (Fig. 4G, H and M). Furthermore, dual hypoxia and serum deprivation significantly decreased LAG-3, A2aR and CD160 on the surface of CD8^+^ T cells compared with cells cultured in cRPMI (Fig. 4G, H and M).

Interestingly, dual hypoxia and glucose deprivation significantly increased A2aR, CTLA-4 and PD-L1 on the surface of CD4^+^ T cells compared with cells cultured in cRPMI (Fig. 4G, H and K). Similarly, dual hypoxia and glucose deprivation significantly increased A2aR, CTLA-4, PD-L1 and PD-L2 on the surface of CD8^+^ T cells compared with cells cultured in cRPMI (Fig. 4G, H, K and L). In contrast, dual hypoxia and glucose deprivation significantly decreased TIGIT expression on the surface of CD8^+^ T cells compared with cells cultured in cRPMI (Fig. 4E).

Additionally, dual glucose and serum deprivation significantly increased PD-1 and decreased KLRG-1 and CD160 on the surface of CD4^+^ T cells compared with cells cultured in cRPMI (Fig. 4D, J and M). Similarly, dual glucose and serum deprivation significantly decreased KLRG-1 and CD160 on the surface of CD8^+^ T cells compared with cells cultured in cRPMI (Fig. 4D and M).

Combined hypoxia with both glucose and serum deprivation significantly increased PD-1 and CTLA-4 on the surface of CD4^+^ T cells compared with cells cultured in cRPMI (Fig. 4D and I). Contrastingly, combined hypoxia with both glucose and serum deprivation significantly decreased CD160 on the surface of CD4^+^ T cells compared with cells cultured in cRPMI (Fig. 4M). Moreover, combined hypoxia with both glucose and serum deprivation significantly increased CTLA-4 and PD-L2 on the surface of CD8^+^ T cells compared with cells cultured in cRPMI (Fig. 4I and L).

Overall, nutrient deprivation and hypoxia had a profound effect on the IC expression profiles of T cells, overall decreasing CD160, TIGIT, LAG-3 and KLRG-1 expression and increasing PD-1 and PD-L1 expression on T cells. Interestingly, depending on the specific combination of conditions of nutrient deprivation and hypoxia, the expression of PD-L2, A2aR and CTLA-4 was differentially expressed on T cell surfaces.

### Hypoxia treatment decreases the frequency of central memory and effector memory T cells and promotes a terminally differentiated state

**Fig. 5:**
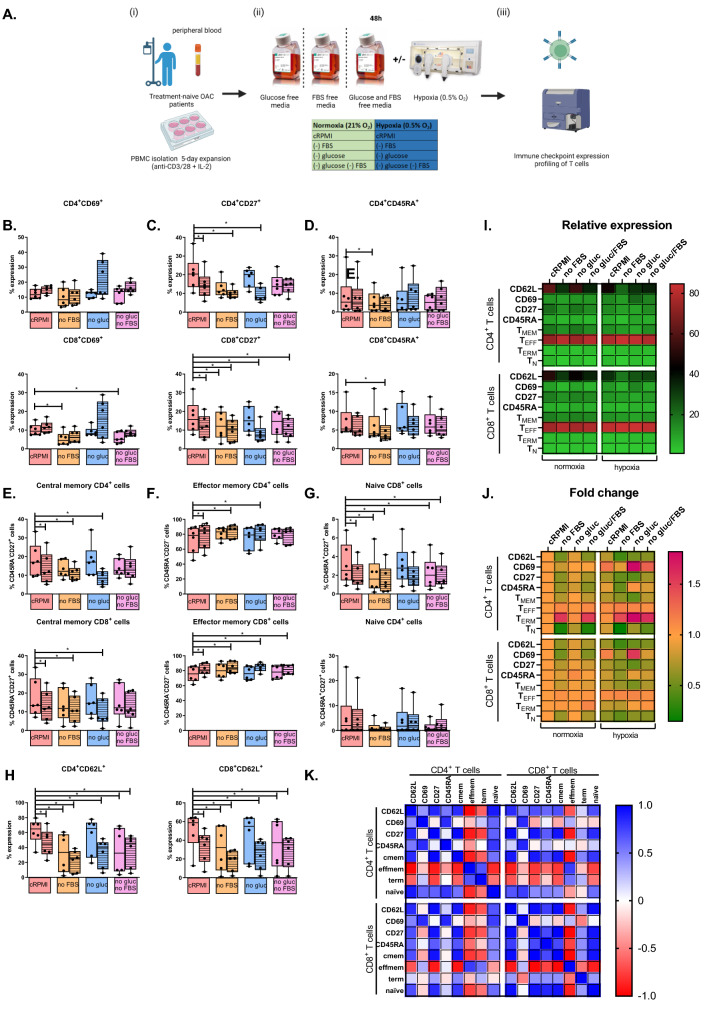
Combination hypoxia and glucose deprivation decreases the frequency of CD27^+^ T cells and central memory T cells while increasing the frequency of effector memory T cells. (**A**) Schematic of experimental setup. PBMCs were isolated from peripheral blood of treatment-naïve OAC patients (*n* = 6) and expanded for 5 days in the presence of plate bound anti-CD3/anti-CD28 and recombinant human IL-2. Following a 5-day expansion, PBMCs were cultured for an additional 24h under nutrient deprivation (FBS deprived or glucose deprived), hypoxia (0.5% O_2_) and combined nutrient deprivation hypoxic conditions. Expression of a range of markers reflective of T cell activation status was assessed on viable CD3^+^CD4^+^ and CD3^+^CD8^+^ cells by flow cytometry. Markers assessed included: CD62L, CD69, CD27 and CD45RA. The percentage of viable naïve (CD45RA^+^CD27^+^), central memory (CDRA-CD27^+^), effector memory (CD45RA-CD27^−^) and terminally differentiated effector memory (CD45RA^+^CD27^−^) CD3^+^CD4^+^ and CD3^+^CD8^+^ (**B**–**H**) cells was also determined by flow cytometry. Undashed bars (left) represent normoxic conditions and dashed bars (right) represent hypoxic conditions. Heat maps that summarise the effect of hypoxia, nutrient deprivation and a combination of both on the relative expression (**I**) and fold change (**J**) on the activation status of CD4^+^ and CD8^+^ cells are also shown. (**K**) Correlation matrix displaying the correlation values for T cell activation marker expression on CD4^+^ and CD8^+^ T cells. Paired, non-parametric *t* test. Expression presented as percentage ± SEM on live cells. Paired, non-parametric t test. Expression presented as percentages ± SEM on live cells, *p < 0.05

To provide further insight into the effect of nutrient deprivation and hypoxia on T cell phenotype, we investigated the effect of nutrient deprivation and hypoxia on T cell differentiation states using OAC patient-derived T cells (Fig. 5).

Hypoxia treatment significantly decreased CD27 and CD62L expression on the surface of CD4^+^ T cells compared with cells cultured under normoxic conditions (Fig. 5C and H). Similarly, hypoxia treatment significantly decreased CD27 and CD62L expression on the surface of CD8^+^ T cells compared with cells cultured under normoxic conditions (Fig. 5C and H). Furthermore, hypoxia treatment significantly decreased the frequency of central memory CD4^+^ T cells (Fig. 5E) and significantly increased the frequency of effector memory CD4^+^ T cells (Fig. 5F) compared with cells cultured under normoxic conditions. Similarly, hypoxia treatment significantly decreased the frequency of naïve CD8^+^ T cells and central memory CD8^+^ T cells and significantly increased the frequency of effector memory CD8^+^ T cells compared with cells cultured under normoxic conditions (Fig. 5E-F).

Serum deprivation significantly decreased CD62L and CD45RA expression on the surface of CD4^+^ T cells compared with cells cultured in cRPMI (Fig. 5H and D). However, serum deprivation did not significantly alter CD4^+^ T cell differentiation states compared with cells cultured in cRPMI (Fig. 5E-G). Similarly, serum deprivation significantly decreased CD27, CD69 and CD62L expression on the surface of CD8^+^ T cells compared with cells cultured in cRPMI (Fig. 5C, B and H). In addition, serum deprivation decreased the frequency of naïve CD8^+^ T cells compared with cells cultured in cRPMI (Fig. 5G).

Interestingly, glucose deprivation alone did not significantly alter the expression of T cell activation markers or frequency of T cell differentiation states compared with cells cultured in cRPMI (Fig. 5).

Dual serum deprivation and glucose deprivation significantly decreased CD62L expression on the surface of CD4^+^ T cells and CD8^+^ T cells compared with cells cultured under cRPMI (Figure H). Furthermore, dual serum deprivation and glucose deprivation significantly decreased CD69 expression on the surface of CD8^+^ T cells compared with cells cultured in cRPMI (Fig. 5B). Dual serum deprivation and glucose deprivation also significantly decreased the frequency of naïve CD8^+^ T cells compared with cells cultured in cRPMI (Fig. 5G).

Combined serum deprivation and hypoxia treatment significantly decreased CD27 and CD62L expression on the surface of CD4^+^ T cells compared with cells cultured in normoxic cRPMI conditions (Fig. 5C and H). Similarly, combined serum deprivation and hypoxia treatment significantly decreased CD27, CD62L and CD45RA expression on the surface of CD8^+^ T cells compared with cells cultured in normoxic cRPMI conditions (Fig. 5C, H and D). Furthermore, combined serum deprivation and hypoxia treatment significantly decreased the frequency of central memory CD4^+^ T cells and subsequently increased the frequency of effector memory CD4^+^ T cells compared with cells cultured in normoxic cRPMI conditions (Fig. 5E and F). Similarly combined serum deprivation and hypoxia treatment significantly decreased the frequency of naïve CD8^+^ T cells and central memory CD8^+^ T cells and increased the frequency of effector memory CD8^+^ T cells compared with cells cultured in normoxic cRPMI conditions (Fig. 5G and E).

Combined glucose deprivation and hypoxia treatment significantly decreased CD27 and CD62L expression on the surface of CD4^+^ T cells compared with cells cultured in normoxic cRPMI conditions (Fig. 5C and H). Combined glucose deprivation and hypoxia treatment significantly decreased the frequency of central memory CD4^+^ T cells and subsequently increased the frequency of effector memory CD4^+^ T cells compared with cells cultured in normoxic cRPMI conditions (Fig. 5E and F). Similar effects were observed in the CD8^+^ T cell compartment, where combined glucose deprivation and hypoxia treatment significantly decreased CD27 and CD62L expression on the surface of CD8^+^ T cells compared with cells cultured in normoxic cRPMI conditions (Fig. 5C and H). Furthermore, combined glucose deprivation and hypoxia treatment significantly decreased the frequency of central memory CD8^+^ T cells and subsequently increased the frequency of effector memory CD8^+^ T cells compared with cells cultured in normoxic cRPMI conditions (Fig. 5E and F).

All three treatments combined, hypoxia with both glucose and serum deprivation significantly decreased CD62L expression on the surface of CD4^+^ T cells compared with cells cultured in normoxic cRPMI conditions (Fig. 5H). Similarly, combined hypoxia with both glucose and serum deprivation significantly decreased CD27 and CD62L expression on the surface of CD8^+^ T cells compared with cells cultured in normoxic cRPMI conditions (Fig. 5C and H). Although combined hypoxia with both glucose and serum deprivation did not significantly affect CD4^+^ T cell differentiation state (Fig. 5). Combined hypoxia with both glucose and serum deprivation significantly decreased the frequency of naive CD8^+^ T cells and subsequently increased the frequency of effector memory CD8^+^ T cells compared with cells cultured in normoxic cRPMI conditions (Fig. 5G and F). Nivolumab has induced durable response rates in only a subpopulation of OAC patients and studies have implicated a role of nutrient depletion and hypoxia for driving resistance to ICB (Power et al. 2020; Chang et al. 2015). Therefore, the ability of nivolumab to enhance T cell activation status or T cell differentiation status was also assessed; however, nivolumab did not significantly alter T cell activation marker expression (Figure S7) or differentiation status of T cells (Figure S8) under nutrient deprivation or hypoxia.

Overall, nutrient deprivation and hypoxia treatment decreased CD27, CD62L and CD45RA expression on T cells and promoted differentiation of CD4^+^ and CD8^+^ T cells into an effector memory-like state, whilst subsequently decreasing the frequencies of naïve and central memory CD4^+^ and CD8^+^ OAC patient-derived T cells.

### Serum deprivation and hypoxia treatment decreased both IL-10 and IFN-γ expression by T cells

**Fig. 6:**
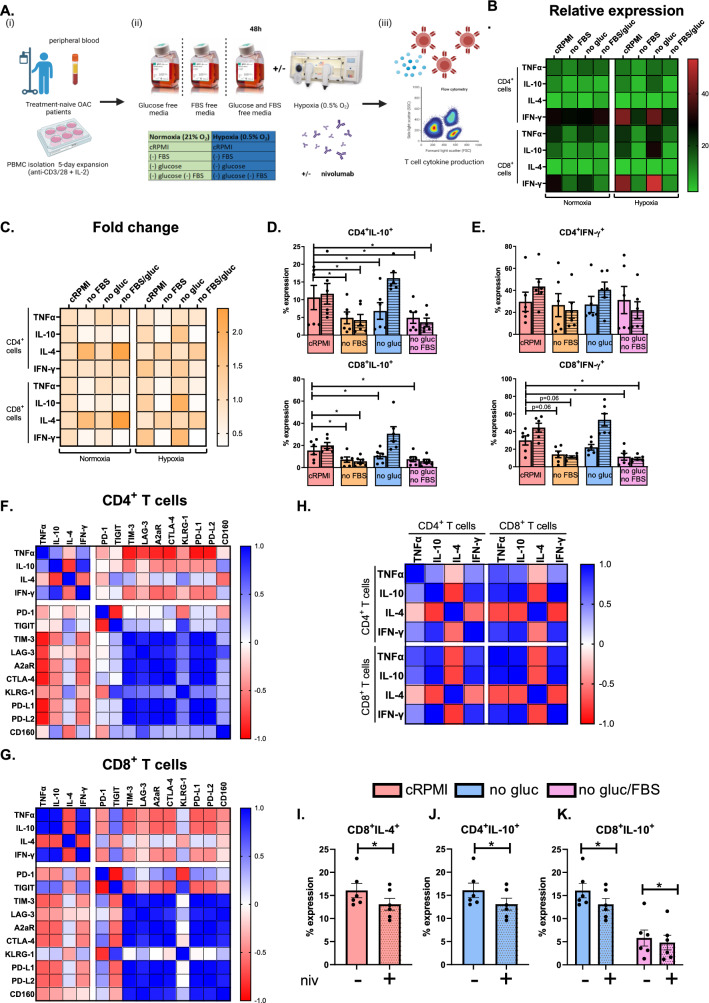
Combination serum deprivation and hypoxia treatment decreases the production of IL-10 and IFN-γ by T cells. (**A**) Schematic of experimental setup. PBMCs were isolated from peripheral blood of treatment-naïve OAC patients (*n* = 6) and expanded for 5 days in the presence of plate bound anti-CD3/anti-CD28 and recombinant human IL-2. Following a 5-day expansion, PBMCs were cultured for an additional 24h under nutrient deprivation (FBS deprived or glucose deprived), hypoxia (0.5% O_2_) and combined nutrient deprivation hypoxic conditions in the absence or presence of nivolumab. Intracellular staining was conducted to assess CD3^+^CD4^+^ and CD3^+^CD8^+^ cell expression of TNF-α, IL-10, IL-4 and IFN-γ by flow cytometry. Paired, non-parametric t test. Expression presented as percentages ± SEM on viable cells. Heat maps that summarise the effects hypoxia, nutrient deprivation and a combination of both on the relative expression (**B**) and fold change (**C**) of cytokine production by CD4^+^ and CD8^+^ cells are also shown. (**D-E**) CD3^+^CD4^+^ and CD3^+^CD8^+^ cell expression of IL-10 and IFN-γ under nutrient deprived and hypoxic conditions was assessed by flow cytometry (n=6). Paired, non-parametric *t* test. Expression presented as percentage ± SEM on live cells. Undashed bars (left) represent normoxic conditions and dashed bars (right) represent hypoxic conditions. (**F**) and (**G**) are correlation matrixes highlighting the correlation values between cytokine production and IC expression in CD4^+^ and CD8^+^ cells. (**H**) Correlation matrix displaying the correlation values for T cell cytokine production by CD4^+^ and CD8^+^ T cells. (**I**–**K**) effect of nivolumab on the production of IL-10 and IL-4 in CD4^+^ and CD8^+^ T cells. Paired, non-parametric t test. expression presented as percentage ± SEM on live cells, *p < 0.05

To determine the effect of nutrient deprivation and hypoxia on OAC T cell phenotypes the expression of Th1-like and Th2-like cytokines were assessed following culture of OAC patient-derived T cells under nutrient deprived and hypoxic conditions for 24 h (Fig. 6).

Hypoxia or nutrient deprivation did not significantly alter the production of TNF-α, IL-4 or IFN-γ by CD4^+^ T cells (Fig. 6). However, the frequency of IL-10 producing CD4^+^ T cells was significantly decreased following 24 h culture under serum deprivation, dual serum deprivation combined with hypoxia, glucose deprivation, dual glucose deprivation and serum deprivation and dual glucose and serum deprivation in combination with hypoxia compared with cells cultured in cRPMI under normoxic conditions (Fig. 6D).

Furthermore, hypoxia or nutrient deprivation did not significantly alter the production of TNF-α or IL-4 by CD8^+^ T cells (Fig. 6B and C). However, the frequency of IL-10 producing CD8^+^ T cells was significantly decreased following 24 h culture under serum deprivation, dual serum deprivation combined with hypoxia, glucose deprivation and dual glucose deprivation and serum deprivation compared with cells cultured in cRPMI under normoxic conditions (Fig. 6D). In addition, the frequency of IFN-γ producing CD8^+^ T cells was significantly decreased following 24 h culture under dual glucose and serum deprivation and dual glucose and serum deprivation in combination with hypoxia compared with cells cultured in cRPMI under normoxic conditions (Fig. 6E). Overall, cytokine production in CD4^+^ T cells largely negatively correlated with IC expression, except in certain instances where IL-4 production positively correlated with TIGIT and KLRG-1 expression and IL-10 production positively correlated with CD160 expression on CD4^+^ T cells (Fig. 6F). Similar results were found in the CD8 compartment. Overall, cytokine production generally negatively correlated with IC expression except on a few occasions in which TNF-α, IL-10, IFN-γ positively correlated with TIGIT expression in CD8^+^ T cells (Fig. 6G).

In addition, the effect of nivolumab on T cell cytokine profiles was also assessed under nutrient deprivation and hypoxia to determine if PD-1 blockade might enhance an anti-tumour phenotype in OAC patient-derived T cells under conditions reflective of a hostile TME.

Interestingly, nivolumab significantly decreased the frequency of IL-10-producing CD4^+^ T cells under combined glucose deprivation and hypoxic conditions compared with untreated cells cultured under those conditions (Fig. 6J). Nivolumab did not significantly affect the frequency of IL-10-producing CD4^+^ T cells under cRPMI conditions, nutrient deprivation alone or hypoxia treatment alone (Figure S9). Similarly, nivolumab significantly decreased the frequency of IL-10-producing CD8^+^ T cells under combined glucose deprivation and hypoxic conditions compared with untreated cells cultured under those conditions (Fig. 6K). Nivolumab also significantly decreased the frequency of IL-10-producing CD8^+^ T cells under combined glucose and serum deprivation under hypoxic conditions compared with untreated cells cultured under those conditions (Fig. 6K). Nivolumab did not significantly affect the frequency of IL-10-producing CD8^+^ T cells under cRPMI conditions, nutrient deprivation alone or hypoxia treatment alone (Figure S9).

Moreover, nivolumab significantly decreased the frequency of IL-4-producing CD8^+^ T cells cultured in cRPMI compared with untreated cells cultured under those conditions (Fig. 6I). However, nivolumab did not significantly affect the frequency of IL-4-producing CD8^+^ T cells under nutrient deprivation or hypoxia treatment alone or combined (Figure S9). In addition, nivolumab treatment did not significantly affect the frequency of IFN-γ or TNF-α expressing T cells cultured in cRPMI, nutrient deprivation, hypoxia treatment or a combination of nutrient deprivation or hypoxia treatment (Figure S9).

Overall, nivolumab decreased the production of IL-10 by T cells under combined glucose deprivation and hypoxia treatment. In addition, nivolumab marginally but significantly decreased the production of IL-4 by T cells cultured in cRPMI but not nutrient deprived or hypoxic conditions.

## Discussion

It is well known that the TME has profound effects on tumour progression and response to treatments by mediating immune suppression (Munn and Bronte 2016). Several mechanisms within the TME are responsible for this and include cellular components such as M2-like macrophages, neutrophils, myeloid-derived suppressor cells, regulatory T cells that produce immunosuppressive factors such as transforming growth factor-β, IL-10, indoleamine 2,3-dioxygenase, arginase, vascular endothelial growth factor, prostaglandins. Collectively these factors promote pro-tumorigenic processes such as angiogenesis, hypoxia and suppression of anti-tumour immunity, which ultimately facilitates immune escape and tumour progression (Gajewski et al. 2006). Importantly, the TME is infamously characterised as a nutrient depleted milieu as a result of rapidly growing tumour cells, which outcompete anti-tumour immune cells for essential nutrients required for their optimal function (Li et al. 2021). The well-known Warburg effect depicts the metabolic hard-wiring of tumour cells to carry out aerobic glycolysis providing tumour cells with an immediate source of fuel but in parallel contributes to rapid depletion of essential nutrient such as glucose which is essential for effector T cell function (Warburg et al. 1927). Similarly, arginase and indoleamine 2,3-dioxygenase selectively deplete arginine and tryptophan, which are essential amino acid required by effector T cells (Mondanelli et al. 2017). Oxygen is also consumed by rapidly growing tumour cells generating hypoxic ‘pockets’ within the tumour and in conjunction with a nutrient depleted TME these features promote angiogenesis and potential metastatic dissemination (Muz et al. 2015). Pro-angiogenic processes, hypoxia and nutrient depletion have profound effects on the metabolism of not just cancer cells but also stromal and tumour-infiltrating immune cells. Such metabolic reprogramming in immune cells ultimately promotes pro-tumorigenic immune cell phenotypes (Li et al. 2021).

Cham et al. demonstrated that glucose deprivation or inhibition of glycolysis by 2-deoxy-D-glucose inhibited the production of IFN-γ, GM-CSF, cytotoxic granule proteins and cell cycle progression  by T cells derived from healthy donors (Cham et al. 2008). Similarly, the findings from this study showed that conditions recapitulating the hostile TME, such as dual glucose and serum deprivation under hypoxic conditions significantly decreased IFN-γ production in OAC patient-derived T cells. In addition to a reduction in IFN-γ production by T cells a decrease in the production of immunosuppressive IL-10 was also observed under nutrient deprivation and hypoxic conditions. This may likely be attributed to the depletion of nutrients which are essential ‘building blocks’ required by T cells to synthesize proteins and subsequent cytokines. Cohen et al., reported that nutrient depletion significantly reduces T cell survival and proliferation and in particular cytokine production (Cohen et al. 2017).

Our results indicate a particularly critical role for glucose in regulating specific effector functions of CD8^+^ T cells. Similarly, our study found that glucose deprivation and serum deprivation, which mimics both a glucose and amino acid deprived TME had the greatest effect in altering T cell activation status and cytokine production, highlighting the immunosuppressive effects of nutrient deprivation within the TME. These findings highlight a key role of a glucose and amino acid depleted TME in driving T cell dysfunction, which likely confers resistance to ICB and other immunotherapies in the OAC setting. These findings also strengthen the rationale for implementing therapeutic approaches that target metabolic restrictions, such as nutrient depletion and hypoxia which have shown promise as combination therapies for different types of cancer to improve T cell metabolic fitness and bolster the anti-tumor immune response (Guo et al. 2019). Glucose consumption by antigenic tumours can metabolically restrict T cells, directly dampening their effector function and allowing tumour progression (Chang et al. 2015). ICB therapy may correct this resource imbalance through a direct effect on tumour cells (Chang et al. 2015). In particular, PD-L1 blockade on melanoma and lung cancer cells inhibited glycolysis resulting in an increased availability of glucose in the TME and subsequently promoted anti-tumour T cell function (Kim et al. 2019).

Interestingly, extremely harsh conditions of combined hypoxia with both glucose and serum deprivation significantly decreased the frequency of naive CD8^+^ T cells and subsequently increased the frequency of effector memory CD8^+^ T cells compared with cells cultured in normoxic cRPMI conditions. It is unclear if these harsh conditions are promoting differentiation of naïve T cells into an effector memory-like state or if these conditions are specifically inducing naïve T cell death and perhaps effector memory-like T cells may be more resilient to these extremely harsh conditions that recapitulate the TME. It has been demonstrated that effector memory-like T cells exhibit high expression levels of anti-apoptotic proteins Bcl-2 (Elyaman et al. 2008) and Mcl-1 (Kim et al. 2016).

Malignant tissue consists not only of tumour cells but also tumour-associated stromal immune cells which are thought to have important roles in tumour growth, disease progression and drug resistance in a context-dependent manner (Yoshihara et al. 2013). An important mechanism by which tumours avoid clearance by the immune system is by inducing the upregulation of inhibitory IC ligands and receptors on tumour cells, immune cells and stromal cells. O’Malley et al. (2018) demonstrated that the soluble inflammatory TME in colorectal cancer induces upregulation of PD-L1 on the surface of stromal cells. PD-L1 expression was identified on both tumour cells and on the immune stroma of 12% and 42% of gastric adenocarcinoma patients undergoing surgical tumour resection, respectively (Thompson et al. 2017). Although higher levels of PD-L1 expression on the tumour cells or immune stroma was associated with an increase in CD8^+^ T cell infiltration, reflecting an ongoing anti-tumour immune response, these patients had reduced progression-free and overall survival. This suggests that PD-L1 plays an important role in mediating immune escape and dampening anti-tumour CD8^+^ effector T cell function (Thompson et al. 2017). Tumour cells upregulate IC ligands on their surface, which negatively regulate T cell activation pathways involved in physiological immune responses against specific antigens, representing significant barriers for induction of effective anti-tumour immune responses. However, the majority of studies to date in OAC focus on assessing the effect of cancer cells on immune cell function and T cell IC expression profiles, while the effect of immune cells on the IC expression profile of cancer cells is often overlooked. Importantly, our study demonstrated that the secreted factors from OAC patient-derived PBMCs, cultured in full nutrient conditions, significantly upregulated PD-L1 and PD-L2 on the surface of OE33 OAC cells in vitro. Similarly, the secretome from OAC patient-derived PBMCs cultured under nutrient deprivation and hypoxia upregulated PD-L1 and PD-L2 on the surface of OE33 cells in vitro. These data suggest that the PD-1 axis may play important role in dampening effector T cell function in OAC by binding to PD-1 on tumour-infiltrating antigen-specific T cells. These findings reaffirm the rationale for administering PD-1 ICB to OAC patients and indicates that the PD-1 axis may play an important role in immune escape. Chen *et al*., performed a meta-analysis for clinical trials testing the efficacy of anti-PD-1 and anti-PD-L1 ICBs in advanced gastric cancers and oesophagogastric cancers, which demonstrated that the addition of ICBs to the second- and third-line settings for treating GOCs improves some, but not all survival endpoints (Chen et al. 2019). The objective response rates were 9.9% and 12.0%, respectively, and the disease control ratios were 33.3% and 34.7%, respectively (Chen et al. 2019). The median progression-free survival (mPFS) was 1.6 months for both ICBs and the median overall survival was 6.0 and 5.4 months, respectively (Chen et al. 2019). Impressive findings from the CheckMate 577 trial demonstrated a substantial therapeutic benefit for administering nivolumab to OAC patients in the adjuvant setting in patients who had residual disease post-surgery in the first-line setting. A doubling in progression-free survival was observed between the nivolumab arm vs. the placebo arm (22 vs. 11 months) (Kelly et al. 2021).

In this study, several ICs were significantly upregulated on the surface of OAC patient-derived T cells following dual glucose deprivation and hypoxia treatment, including PD-1, PD-L1, PD-L2 and CTLA-4 ICs. IC intrinsic signalling in T cells has profound effects on T cell metabolism, PD-1 intrinsic signalling in T cells inhibits glycolysis and promotes lipolysis and fatty acid oxidation (Patsoukis et al. 2015), similarly, CTLA-4 intrinsic signalling in T cells inhibits glycolysis (Patsoukis et al. 2015). Glycolysis is essential for effector T cell function; therefore, upregulation of PD-1 and CTLA-4 could be detrimental to anti-tumour immunity and may reflect the skewing of T cells toward an altered phenotype facilitated by PD-1 and CTLA-4 metabolic reprogramming. However, a fatty acid oxidative phenotype that could be promoted by PD-1 and CTLA-4 signalling is utilised by both regulatory T cells (Raud et al. 2018) and tissue resident memory T cells, which are known for their anti-tumour functions (Lin, et al. 2020). CTLA-4 expression on regulatory T cells plays a pivotal role in hindering anti-tumour immunity by promoting regulatory T cell function, which suppresses antigen-presenting cells by depleting immune stimulating cytokines, producing immunosuppressive cytokines and constitutively expressing CTLA-4 (Sobhani 2021). Preclinical murine models have demonstrated that CTLA-4 blockade promotes cancer regression by increasing the frequency of effector T cells within the TME and selectively depleting intra-tumoral regulatory T cells via an Fc-dependent mechanism (Sharma et al. 1233).

In contrast, certain conditions also decreased specific ICs, dual hypoxia and serum deprivation significantly decreased LAG-3, A2aR and CD160 on the surface of CD8^+^ T cells compared with cells cultured in cRPMI and dual hypoxia and glucose deprivation significantly decreased TIGIT expression on the surface of CD8^+^ T cells compared with cells cultured in cRPMI. These particular conditions are very harsh and may be a reflection of reduced protein synthesis, which subsequently translated into a reduction in the production and surface expression of IC proteins.

Interestingly, this study demonstrated that nivolumab treatment significantly reduced the production of IL-10 by T cells under glucose deprived hypoxic conditions compared with untreated cells under glucose deprived hypoxic conditions, suggesting that PD-1 blockade may help skew T cells toward an anti-tumour phenotype within the OAC TME. However, nivolumab did not decrease IL-10 production by T cells under complete nutrient conditions, highlighting that nivolumab was more effective at promoting an anti-tumour T cell phenotype under ‘stressful’ glucose deprived and hypoxic conditions which are more reflective of the TME than full nutrient conditions.

Surprisingly, nivolumab significantly decreased the surface expression of CD69 on the surface of T cells under glucose deprived hypoxic conditions compared with untreated cells under glucose deprived hypoxic conditions. CD69 is an important co-stimulatory molecule that sustains T cell activation, proliferation and cytolytic activity (Ma et al. 2017). However, studies have implicated a role for CD69 in promoting exhaustion in tumour-infiltrating T cells perhaps as a result of prolonged T cell activation promoted by CD69 signalling in the T cells (Blackburn et al. 2009). Mita et al. demonstrated in *CD69*^*–/–*^ mice using 4T1-luc2 murine breast cancer models, a significant reduction in tumour growth and metastasis, increased levels of tumour-infiltrating lymphocytes and a significant reduction in T cell exhaustion and enhanced IFN-γ production compared with wild-type controls (Blackburn et al. 2009). Additionally, anti-CD69 monoclonal antibody treatment attenuated the T-cell exhaustion and tumour progression in tumour-bearing mice (Blackburn et al. 2009). Considering our findings in the context of the study by Mita et al. (Blackburn et al. 2009), nivolumab-induced downregulation of CD69 under glucose deprived hypoxic conditions may be a benefit to anti-tumour T cell function. Furthermore, nivolumab has currently been FDA approved for the treatment of OAC and several other IC inhibitors targeting the PD-1 axis and CTLA-4 axis are currently in clinical trials (Davern and Lysaght 2020; Smyth and Thuss-Patience 2018).

However, tumour cells are able to counteract the activity of PD-1 and CTLA-4 ICBs and can commission additional inhibitory pathways via expression of other ICs/ligands within the TME (Lee et al. 2021). Of particular clinical relevance regarding mechanisms for development of acquired resistance to therapeutics targeting the conventional PD-1 and CTLA-4 axes, the A2aR IC receptor was also upregulated on the surface of T cells under nutrient deprivation and hypoxic conditions. Furthermore, co-culturing OAC cells with PBMCs upregulated LAG-3 on the surface of T cells. These novel ICs have been shown to have profound immunosuppressive effects on effector T cells. Therefore, co-blockade of multiple ICs may be a better strategy to enhance effector T cell function in OAC. Clinical trials are ongoing in other cancer types targeting LAG-3 and A2aR (Braun et al. 2021). A2aR elicits profound immunosuppressive effects within the TME. Regulatory T cells secrete adenosine within the TME, which potently inhibits production of IFN-γ, IL-1, IL-2, IL-3, IL-4, IL-12, IL-13, TNF-α, granulocyte–macrophage colony-stimulating factor, CCL3, and CCL4 in effector T cells upon binding to its cognate receptor A2aR (Romio et al. 2011). Increased expression of A2aR on tumour infiltrating immune cells correlated with advanced pathological grade, larger tumour size and positive lymph node status in head and neck squamous cell carcinoma (HNSCC) (Ma et al. 2017). Interestingly, the expression of A2AR was found to significantly correlate with HIF-1α, CD73, CD8 and Foxp3. Furthermore, the increased population of CD4^+^Foxp3^+^ regulatory T cells (Tregs), which partially expressed A2aR, was observed in an immunocompetent mouse model that spontaneously develops HNSCC (Ma et al. 2017). Pharmacological blockade of A2aR by SCH58261 delayed the tumour growth in the HNSCC mouse model and significantly decreased the population of CD4^+^Foxp3^+^ Tregs and enhanced the anti-tumour response of CD8^+^ T cells (Ma et al. 2017). These studies highlight the important role A2aR plays in the TME for promoting tumour progression and the pharmacologic impact of A2aR inhibition for promoting anti-tumour responses.

Similarly, LAG-3 is expressed on the surface of activated T cells and is emerging as an important IC in supressing several arms of the anti-tumour repertoire of immune cells and is garnering a lot of attention as a therapeutic target to reinvigorate exhausted T cells (Blackburn et al. 2009). Grosso et al. (2007), also demonstrated that LAG-3 knockout adoptively transferred antigen-specific CD8 T cells in mice bearing their cognate antigen, as either a self or a tumour antigen, showed enhanced proliferation and cytokine production. Furthermore, expression of LAG-3 on regulatory CD4 T cells identified a more immunosuppressive phenotype, which subsequently hindered CD8 T cell function in Hodgkin’s lymphoma (Gandhi et al. 2006), melanoma and colorectal cancer (Camisaschi et al. 2010). Collectively, these studies identify a pivotal role for LAG-3 in dampening anti-tumour immunity and highlight that LAG-3 blockade can induce durable responses in pre-clinical models. Of particular clinical relevance for designing combination ICB therapies, co-targeting LAG-3 in combination with PD-1 inhibition is thought to achieve synergistic responses. To date at least 13 agents that target LAG-3 have been developed and are under clinical trials for various cancers (Maruhashi et al. 2020). In phase I/II study evaluating the safety and efficacy of relatlimab (anti-LAG-3) in combination with nivolumab in patients with advanced melanoma that had progressed during previous anti-PD-1 or anti-PD-L1 immunotherapy (NCT0198609), the combination of relatlimab and nivolumab was well tolerated and the objective response rate (ORR) was 11.5% in 61 patients. ORR was at least 3.5-fold higher in patients with LAG-3 expression in at least 1% of tumour-associated immune cells within the tumor margin (*n* = 33) than that in the patients with less than 1% LAG-3 expression (*n* = 22) (18% and 5%, respectively) (Ascierto et al. 2017). Similarly, LAG525 in combination with anti-PD-1 (spartalizumab) exhibited a durable response in 9.9% of patients (*n* = 121) with a variety of solid tumors, including mesothelioma (two of eight patients) and triple-negative breast cancer (two of five patients) in phase I/II study (NCT02460224) (Hong et al. 2018).

Taken together, these pre-clinical and clinical studies investigating A2aR and LAG-3 demonstrate that targeting these novel ICs to enhance anti-tumour immunity is a viable strategy for boosting the efficacy of conventional PD-1 ICBs. However, A2aR and LAG-3 IC pathways remain under-investigated in the context of OAC and acquiring a deeper insight into the expression profiles of ICs in OAC patients and understanding how features of the hostile TME affect IC expression profiles will help guide rational therapeutic design of appropriate immunotherapeutic strategies to overcome features of the immunosuppressive TME in OAC patients.

Overall, this study highlights that multiple ICs are expressed on circulating immune cells and tumour-infiltrating stromal immune cells. In addition, nutrient deprivation had the greatest effect on upregulating several ICs on T cell surfaces, which likely cooperate in tandem to suppress anti-tumour immunity within the TME.

## Supplementary Information

Below is the link to the electronic supplementary material.

Supplementary file1 (DOCX 1916 KB)

## Data Availability

No datasets were generated from this study and clinical data cannot be made available.
