# Supplementary material for: Nutrient deprivation and hypoxia alter T cell immune checkpoint expression: potential impact for immunotherapy
Source: J Cancer Res Clin Oncol. 2022 Nov 29;149(8):5377–95. doi: 10.1007/s00432-022-04440-0 (PMC10349772; doi:10.1007/s00432-022-04440-0)
Supplement: Supplementary file 1 — Supplementary file1 (DOCX 1916 KB) [file 432_2022_4440_MOESM1_ESM.docx]

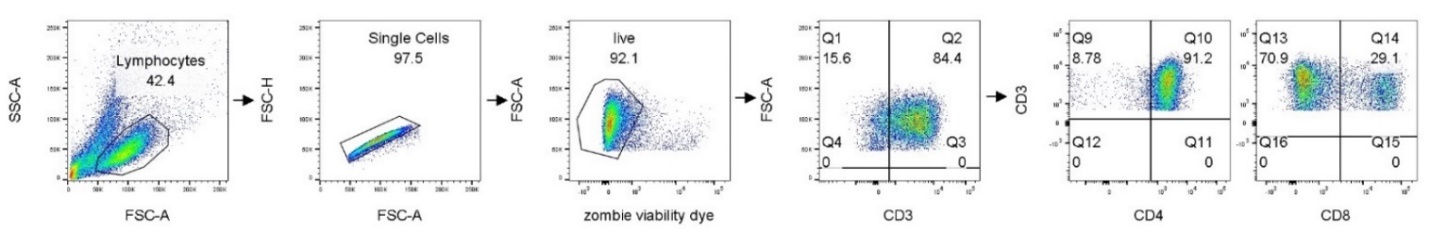

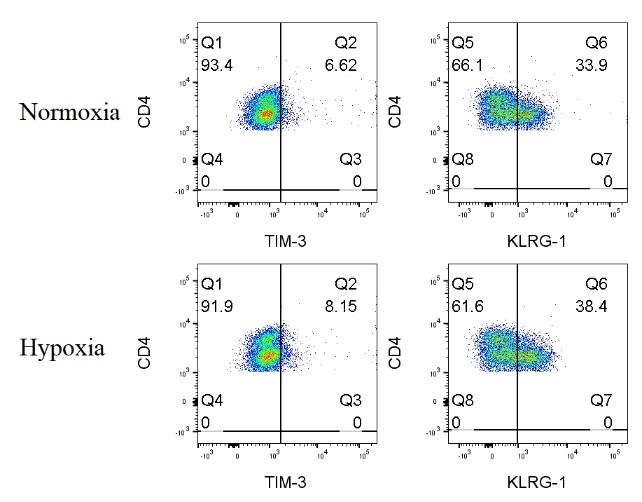

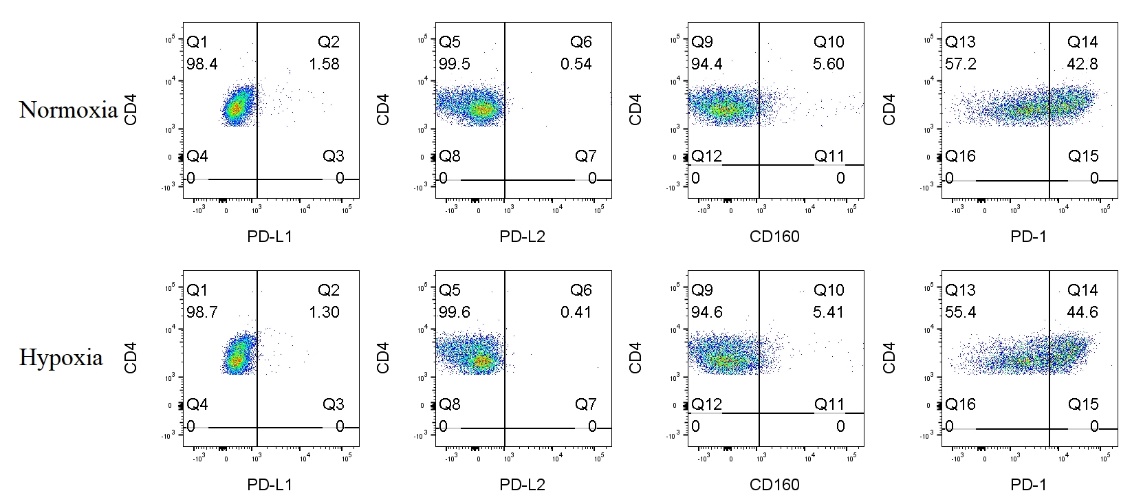
Supplemental


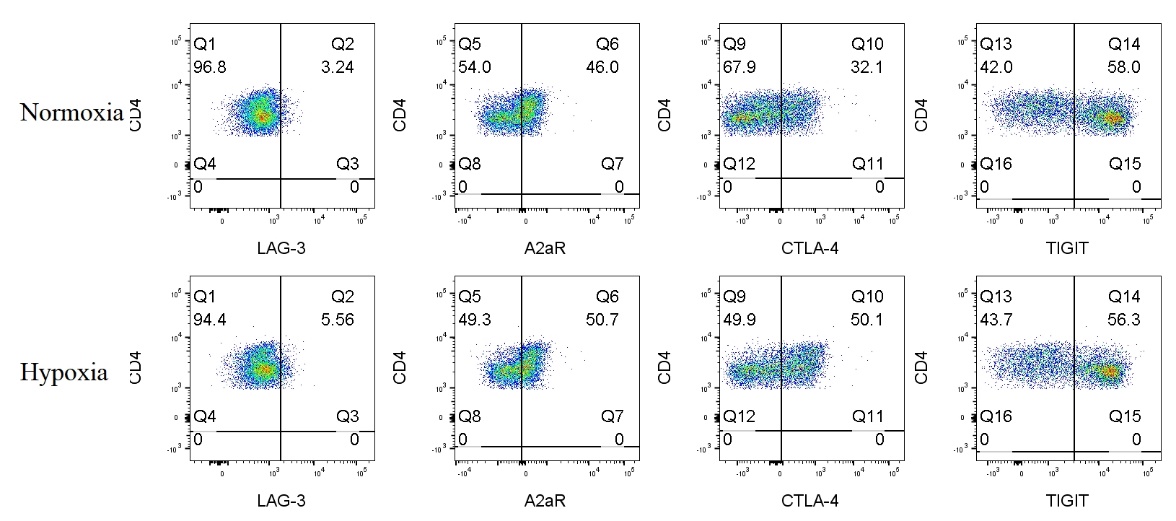


**Figure S1:** **Gating strategy for assessing expression of ICs on T cells surfaces by flow cytometry.** Gate 1 is the lymphocyte gate which included all cells in the FSC versus SSCA plot, doublet cells were then excluded using FSC-H versus FSC-A plot, dead cells were excluded using zombie viability dye. The surface expression of PD-L1, PD-L2, CD160, PD-1, TIM-3, KLRG-1, LAG-3, A2aR, CTLA-4 and TIGIT was assessed on CD3^+^CD4^+^ cells and CD3^+^CD8^+^ cells. Representative dot plots are shown for each gated on CD3^+^CD4^+^ cells following 48h culture of PBMCs under normoxia and hypoxia.


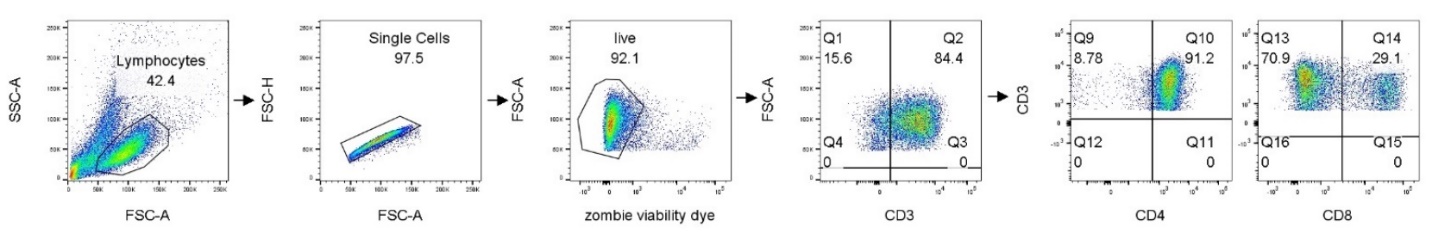


**
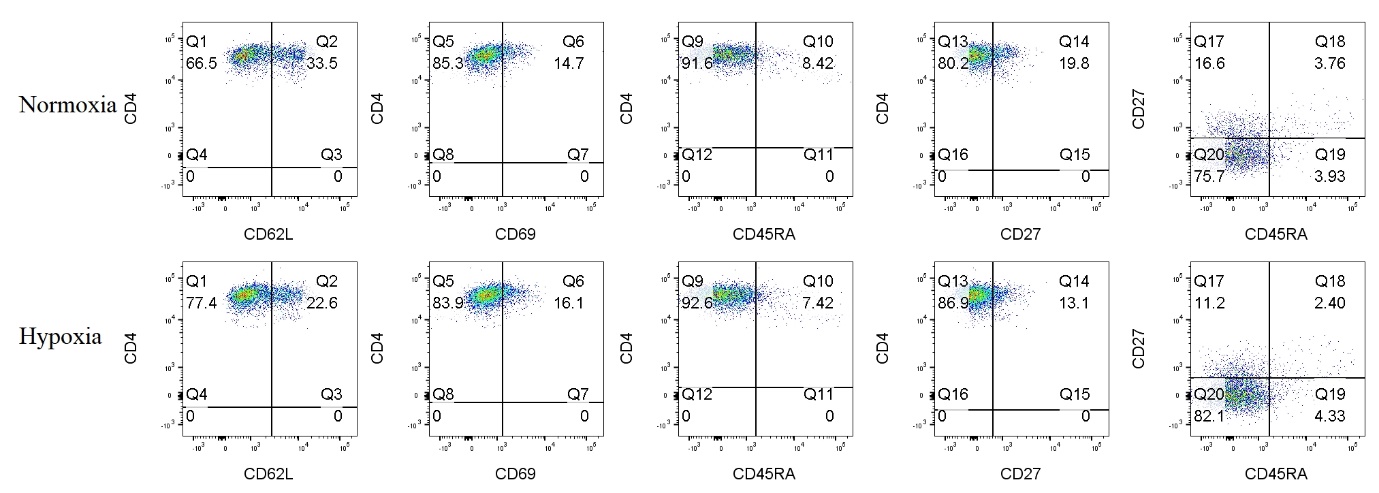
**

**Figure S2: Gating strategy for assessing expression of T cell activation markers and T cell differentiation states by flow cytometry.** Gate 1 is the lymphocyte gate and included all cells in the FSC versus SSCA plot, doublet cells were then excluded using FSC-H versus FSC-A plot, dead cells were excluded using zombie viability dye. The surface expression of CD62L, CD69, CD45RA and CD27 as well as T cell differentiation states was assessed on CD3^+^CD4^+^ cells and CD3^+^CD8^+^ cells. Representative dot plots are shown for each marker gated on CD3^+^CD4^+^ cells following 48h PBMCs under normoxia and hypoxia. Representative dot plots also shown depicting viable naïve (CD27^+^CD45RA^+^), central memory (CD27^+^CD45RA^-^), effector memory (CD27^-^CD45RA^-^) and terminally differentiated effector memory (CD27^-^CD45RA^+^) CD3^+^CD4^+^ cells.


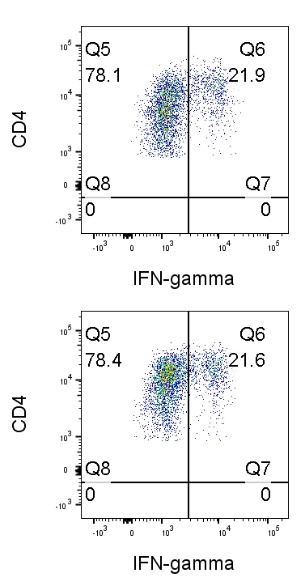

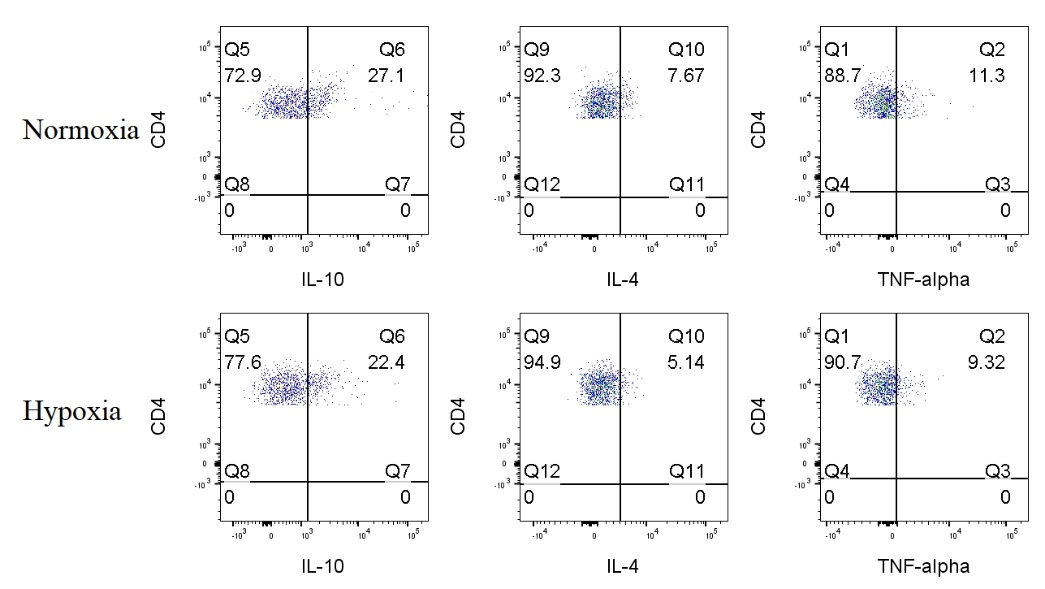

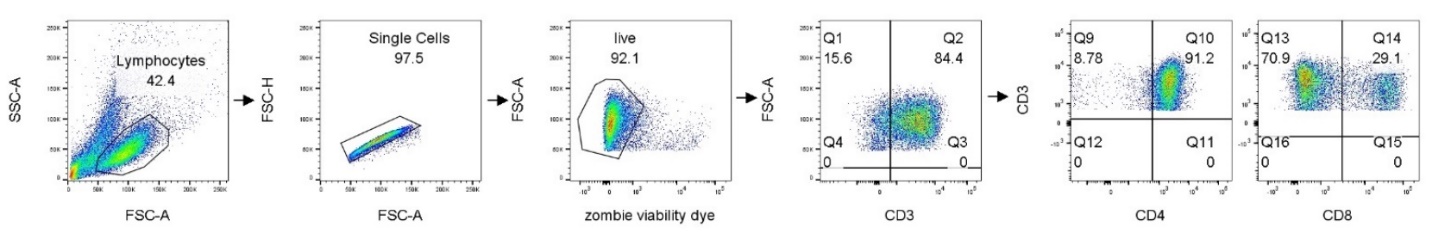


**Figure S3: Gating strategy for assessing cytokine production by T cells by flow cytometry.** Gate 1 is the lymphocyte gate and included all cells in the FSC versus SSCA plot, doublet cells were then excluded using FSC-H versus FSC-A plot, dead cells were excluded using zombie aqua viability marker. The intracellular surface expression of IL-10, IL-4, TNF-α, and IFN-γ was assessed on CD3^+^CD4^+^ cells and CD3^+^CD8^+^ cells. Representative dot plots are shown for IL-10, IL-4, TNF-α, and IFN-γ gated on CD3^+^CD4^+^ cells following 48h culture of PBMCs under normoxia and hypoxia.

**Figure S4. Co-culturing OE33 cells with OAC donor PBMCs did not significantly alter the expression of PD-1, TIGIT, TIM-3, A2aR, CTLA-4, PD-L1, PD-L2 and CD160 on the surface of T cells.** OE33 cells were cultured for 24h in the absence or presence of PBMCs (200,000 OE33: 400,000 PBMCs) isolated from OAC patients (n=7) that were pre-activated for 5 days with plate bound anti-CD3/28 and IL-2 prior to culture. CD3^+^, CD3^+^CD4^+^ and CD3^+^CD8^+^ cells were then stained with a zombie viability dye and antibodies specific for a range of inhibitory immune checkpoint receptors (PD-1, TIGIT, TIM-3, A2aR and CTLA-4) and inhibitory immune checkpoint ligands (PD-L1, PD-L2 and CD160) and expression was assessed by flow cytometry. Paired, non-parametric t test. Expression presented as percentage ± SEM on live cells.

**Figure S5. Co-culturing OE33 cells with OAC donor lymphocytes did not significantly alter the expression of CD62L or CD45RO on the surface of T cells.** OE33 cells were cultured for 24h in the absence or presence of PBMCs (1:2 OE33: PBMCs) isolated from OAC patients (n=7) that were pre-activated for 5 days with plate bound anti-CD3/28 and IL-2 prior to culture. Expression of CD62L and CD45RO were assessed on viable CD3^+^, CD3^+^CD4^+^ and CD3^+^CD8^+^ cells by flow cytometry. Dead cells were excluded using a zombie viability dye. Paired, non-parametric t test. Expression presented as percentage ± SEM on live cells.

**Figure S6. Co-culturing OE33 cells with OAC donor lymphocytes did not significantly alter the frequency of IL-17A/F^+^, IFN-γ^+^, TNF-α^+^, IL-4^+^ and IL-10^+^ T cells or IL-10^+^ T cells.** OE33 cells were cultured for 24h in the absence or presence of PBMCs (1:2 OE33: PBMCs) isolated from OAC patients (n=6) that were pre-activated for 5 days with plate bound anti-CD3/28 and IL-2 prior to culture. Intracellular staining was conducted to assess CD3^+^, CD3^+^CD4^+^ and CD3^+^CD8^+^ cell production of IL-17A/F, IFN-γ, TNF-α, IL-4 and IL-10 cytokines by flow cytometry. Paired, non-parametric t test. Expression presented as percentage ± SEM on live cells.

**Figure S7.** **Nivolumab significantly decreased CD69 expression on T cells under nutrient deprivation.** PBMCs were isolated from peripheral blood of treatment-naïve OAC patients (n=6) and expanded for 5 days in the presence of plate bound anti-CD3/anti-CD28 and recombinant human IL-2. Following a 5-day expansion, PBMCs were cultured for 24h under nutrient deprivation (FBS deprived or glucose deprived), hypoxia (0.5% O_2_) and combined nutrient deprivation hypoxic conditions in the absence or presence of nivolumab (niv). Expression of a range of markers reflective of T cell activation status was assessed on viable CD3^+^CD4^+^ (A) and CD3^+^CD8^+^ (B) cells by flow cytometry (n=3). Markers assessed included: CD62L, CD69, CD27 and CD45RA. Dead cells were excluded using a zombie viability dye. Paired, non-parametric t test. Expression presented as percentages ± SEM on live cells. Abbreviations: (-): no nivolumab, (+): nivolumab (10 μg/ml), no gluc: no glucose, N: normoxia, H: hypoxia.

**Figure S8. Nutrient deprivation and hypoxia substantially alter T cell differentiation status, however addition of nivolumab did not significantly affect T cell differentiation state under these conditions.** PBMCs were isolated from peripheral blood of treatment-naïve OAC patients (n=6) and expanded for 5 days in the presence of plate bound anti-CD3/anti-CD28 and recombinant human IL-2. Following a 5-day expansion, PBMCs were cultured for 24h under nutrient deprivation (FBS deprived or glucose deprived), hypoxia (0.5% O_2_) and combined nutrient deprivation hypoxic conditions in the absence or presence of nivolumab (niv). The percentage of viable naïve (CD45RA^+^CD27^+^), central memory (CDRA^-^CD27^+^), effector memory (CD45RA^-^CD27^-^) and terminally differentiated effector memory (CD45RA^+^CD27^-^) CD3^+^CD4^+^ (A) and CD3^+^CD8^+^ (B) cells was determined by flow cytometry. Dead cells were excluded using a zombie viability dye. Paired, non-parametric t test, *p<0.05. Expression presented as percentages ± SEM on live cells. Abbreviations: (-): no nivolumab, (+): nivolumab (10 μg/ml), no gluc: no glucose, N: normoxia, H: hypoxia.

**Figure S9. Nutrient deprivation and hypoxia substantially altered IFN-γ production by T cells however addition of nivolumab under these conditions did not significantly alter the cytokine production of TNF-α or IFN-γ by T cells or cytotoxic potential.** PBMCs were isolated from peripheral blood of treatment-naïve OAC patients (n=6) and expanded for 5 days in the presence of plate bound anti-CD3/anti-CD28 and recombinant human IL-2. Following a 5-day expansion, PBMCs were cultured for an additional 24h under nutrient deprivation (FBS deprived or glucose deprived), hypoxia (0.5% O_2_) and combined nutrient deprivation hypoxic conditions in the absence or presence of nivolumab. Intracellular staining was conducted to assess CD3^+^CD4^+^ (A) and CD3^+^CD8^+^ (B) cell production of TNF-α and IFN-γ cytokines by flow cytometry. Cytotoxic potential was also assessed by a CD107a degranulation assay in CD8^+^ T cells by flow cytometry (B). Paired, non-parametric t test. Expression presented as percentages ± SEM on viable cells. Abbreviations: (-): no nivolumab, (+): nivolumab (10 μg/ml), no gluc: no glucose, N: normoxia, H: hypoxia.
